# Deep-Sea Nematodes Actively Colonise Sediments, Irrespective of the Presence of a Pulse of Organic Matter: Results from an In-Situ Experiment

**DOI:** 10.1371/journal.pone.0018912

**Published:** 2011-04-19

**Authors:** Katja Guilini, Thomas Soltwedel, Dick van Oevelen, Ann Vanreusel

**Affiliations:** 1 Department of Biology, Marine Biology Section, Ghent University, Ghent, Belgium; 2 Department of Deep Sea Ecology and Technology, Alfred Wegener Institute, Bremerhaven, Germany; 3 Centre for Estuarine and Marine Ecology, Netherlands Institute of Ecology (NIOO-KNAW), Yerseke, The Netherlands; Institute of Marine Research, Norway

## Abstract

A colonisation experiment was performed in situ at 2500 m water depth at the Arctic deep-sea long-term observatory HAUSGARTEN to determine the response of deep-sea nematodes to disturbed, newly available patches, enriched with organic matter. Cylindrical tubes,laterally covered with a 500 µm mesh, were filled with azoic deep-sea sediment and ^13^C-labelled food sources (diatoms and bacteria). After 10 days of incubation the tubes were analysed for nematode response in terms of colonisation and uptake. Nematodes actively colonised the tubes,however with densities that only accounted for a maximum of 2.13% (51 ind.10 cm^−2^) of the ambient nematode assemblages. Densities did not differ according to the presence or absence of organic matter, nor according to the type of organic matter added. The fact that the organic matter did not function as an attractant to nematodes was confirmed by the absence of notable ^13^C assimilation by the colonising nematodes. Overall, colonisationappears to be a process that yields reproducible abundance and diversity patterns, with certain taxa showing more efficiency. Together with the high variability between the colonising nematode assemblages, this lends experimental support to the existence of a spatio-temporal mosaic that emerges from highly localised, partially stochastic community dynamics.

## Introduction

Vast deep-sea soft-sediment areas that appear to be static and monotonous are in fact subject to a variety of natural disturbances. The spatial extent of disturbance and the temporal scales at which disturbed patches are produced range from small-scale, often ephemeral tracks, pits, tubes and faecal mounds,through intermediate-scaleseasonal phytodetritus falls,benthic storms and dense water cascadingevents, up to large-scale events like turbidity storms or landslides [1], [2]. These disturbances create a spatial and temporal mosaic in whichmodified conditions and often newly available habitats provide the opportunity for colonisation [3]. According to the patch-mosaic model, each of these patches evolves separately through a succession process driven by varied forms of biotic forcing(colonisation ability, reproductive success, growth efficiency, biological interactions, niche diversification, larval dispersal capacity, and other life strategies), and as such enhances the coexistence of a large number of species [4]. To verify the model and to understand the processes that structure the highly diverse benthic deep-sea community, basic knowledge on rates and patterns of faunal colonisation and succession is crucial [5].

Perhaps the most important factor structuring the soft-sediment community is the spatio-temporal input of organic matter, originating from the phototrophic surface of the oceans [6], [7]. The input can beconsidered as a disturbance due to its seasonal and patchy character, but at the same time as the energy source that sustains the benthic community [8], [9]. Bacteria, Protozoa and several macrobenthic taxa have shown a rapid natural response [10] and considerable uptake of phytodetritus in ^13^C-tracer experiments that simulated pulses of phytodetritus [11], [12]. Instead, the reaction of the deep-sea metazoan nematodes is relatively slow [7] and uptake of ^13^C-labelled phytodetritus was always limited [12], [13]or absent [14]–[16]. This implies that nematodesthat dominate abundance, biomass and probably local species richness amongst the metazoan meiofauna [17], [18], display a trophic specialisation and/or slow biological processes that avoid competitive displacement [19]. To understand the success of deep-sea nematode communities, it is necessary to first investigate their ecology and the principles of succession processes, starting with the early arrival of species in newly available patches.

Colonisation experiments have been used widely to study the impact of disturbance, and in particular food deposition, on benthic community structures ([20] and references therein). Mobile macrofauna in both deep and shallow areas are rapidly attracted to experimentally deposited food. As such, macrofaunal colonisation of food-enriched patches generally results in initially higher abundances and lower diversity compared to the ambient sediment(e.g. [9], [21]–[23]).Nematodes may also recolonise and reach background densities within hours or days after disturbance [24]–[26]. However, this has only been demonstrated in shallow and intertidal sediments where wave turbulence and tidal currents are strong enough to enable resuspension and passive distribution of nematodes through the water column. Nematode colonisation in deep and low energy environments is not stimulated by physical transport, but instead relies predominantly on active migration by nematodes. Nematodes with a large body size can more easily burrow through the sediment [27] and might therefore have greater colonisation abilities via infaunal migration compared to smaller congeners [28], [29]. Gallucci et al. [28] performed an on-board microcosm study to investigate active colonisation of defaunated Arctic sediments from 1300 m water depth. The defaunated sediments remained unenriched or were enriched with the diatom *Thalassiosira weissflogii*(1 g organic C m^−2^). Nematodes colonised both sediments with mean abundances corresponding to5% (unenriched) and 20% (enriched) ofmean nematode abundances in the controls after 9 days. Nematode abundance was significantly higher in the enriched sediments, suggesting fresh detritus enhances colonisation and/or resilience. Colonisation rates measured in this experiment were similar to, or at the lower end of reports on small-scale infaunal migration into azoic sediments by nematodes in subtidal areas at comparable time-scales [30]–[34]. However, the majority of the colonising species were rare or undetected members of the background sediments and the colonising community differed strongly between the defaunated sediments. The significantly higher colonisation of the enriched sediments seems to be in contrast with the results from the ^13^C-phytodetritus experiments, where uptake rates of deep-seanematodes are consistently low (see above). However, given that mostly rare species responded with rapid colonisation in the experiment by Gallucci et al. [28], their uptake in the ^13^C-phytodetritus experiments may have been masked by the majority of less responsive nematodes.

To elucidate the importance of nematode infaunal migration in determining small-scale temporal and spatial heterogeneity and the role of organic matter deposits in this process in the deep sea, this study combined measurements of nematode colonisation and food-uptake. More specific, we compare the short-term, in-situ response of deep-sea nematodes to different pulses of organic matter in terms of colonisation and food uptake.The response was monitored by inserting experimental tubes into the sediment at 2475 m water depth by means of ROV manipulation. The tubes were laterally covered with a 500 µm mesh and containedazoic sediments. In addition to undisturbed sediment, four ^13^C-labelled potential food sources were used: 1) thediatom *Thalassiosira pseudonana*, 2) thediatom *Skeletonema costatum*, 3) benthic bacteria and 4) bacteria grown on degrading diatoms (only bacteria were labelled, see below). Diatoms were selected to determine colonisation of patches with settled labile phytodetritus, associated with medium scale disturbance. Bacteria are attached and involved in the decomposition of settling particulate organic matter, and were therefore also considered as a potentially favoured food source.Benthic bacteria were chosen as they represent a more stable background resource, with constant biomass and abundance in marine sediments [35]. The following hypotheses were tested:

(H_1_) Deep-sea nematodes colonise disturbed, newly available habitats.

(H_2_) The presence of fresh food enhances the rate of colonisation and is reflected in higher nematode abundances and numbers of coexisting genera.

(H_3_) Mainly rare nematode species colonise the experimental, enriched tubes and food consumption is evident in strong isotope enrichment as compared to earlier ^13^C-phytodetritus experiments that monitored the response of the bulk community.

(H_4_) The colonising nematodes show preferencefor an added type of food.

(H_5_) Active migration is species-specific, i.e. some species may be more efficient than others and therefore, colonisation might not be entirely random.

## Materials and Methods

### Study site and sampling

**Figure 1:**
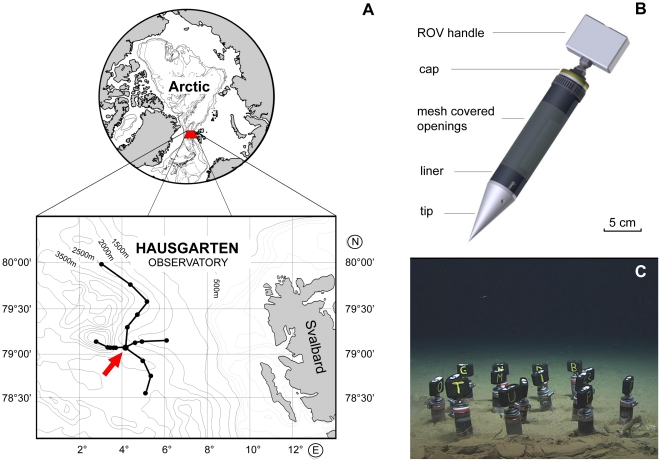
Location of the study area at the Arctic Marginal Ice Zone in the Fram Strait (Greenland Sea), west of Svalbard; a detailed design of the colonisation tubes and a close-up of the experimental site at 2475 m water depth. A: Bathymetric map of the study area, with a dot at every HAUSGARTEN station. The arrow marks the central HAUSGARTEN station (HG IV) where the experiment was conducted. B: Colonisation tube design with indicative scale. C: The experimental plot at 2475 m water depth with randomly distributed colonisation tubes. Imagetaken by ROV *Quest*. Image copyright by MARUM, University of Bremen.

The experiment was conducted at 2475 mwater depth at the central HAUSGARTEN station (HG IV, Figure 1A) in the Fram Strait (west of Svalbard, 79°04.7′N, 4°05.7′E) during the ARK XXII-1c campaign in July 2007. HAUSGARTEN is a long-term observatory established in 1999 by the German AlfredWegener Institute for Polar and Marine Research [36].The remotely operated vehicle (ROV) *Quest* (MARUM, University of Bremen) carried out the video-controlled deployment and recovery of the experimental tubes. Reference samples, to determine the background nematode community composition, were collected from a single multi-corer (MUC) deployment conducted in close vicinity to the experimental site(July 2007, 79°03.9′N, 4°10.6′E, 2.2 km from the experimental plot). Three (pseudo)replicates were subsampled with syringes (2 cm diameter), sliced per centimetre down to a depth of 5 cm and fixed in 4% formalin. One push core from the ROV operations was taken, sliced per 5 cm to a depth of 15 cm and stored frozen at −20°C to determine the background nematode ^13^C values, total organic carbon content (TOC) in the sediment and grain-size distribution. The sediment from below 15 cm depth in a box corer served as azoic sediment to fill the experimental colonisationtubes and was also sampled with syringes for background nematode abundance, grain-size information and TOC of the sediment (3-times 3 syringes grouped together; 2 cm diameter, 5 cm length).

### Cultivation and ^13^C enrichment of food sources

Prior to the experiment, the cosmopolitan marine, centric diatoms *Thalassiosira pseudonana* (centric, singular, cell radius 3 µm)and *Skeletonema costatum* (cylindrical, filamentous, cell radius 3.125 µm) were both cultured axenically in f2 medium [37] at 15°C with a 12∶12-h light-dark period. Diatoms were enriched by adding 5 ml of aNaH^13^CO_3_ MilliQ solution (^13^C, 99%, Cambridge Isotope Laboratories, 336 mg per 100 ml MilliQ H_2_O)to 100 ml of the culture medium. After approximately 14 days, the algal material was harvested by centrifugation (3500 rpm, 5 min) and rinsed 3-times with artificial seawater to remove remaining bicarbonate ^13^C from the algal suspension. The axenic state of the algae was verified by microscopic observations. This labelling technique resulted in an average δ^13^C value of 43,259±1,920‰ (equalling 33% ^13^C) and 55,311±675‰ (equalling 39% ^13^C) for *T. pseudonana* and *S. Costatum*,respectively.

In order to mimic a degrading algal bloom with an associated microbial community,*T. pseudonana* was also cultured non-axenically (i.e. non-sterilised conditions) under similar cultivation conditions as described above, but instead non-labelled NaHCO_3_was addedto the f2 medium. To stimulate the growth and isotopically enrich the bacteria in the non-axenic culture, the algae were placed in the dark after 14 days of incubation and both unlabelled D-glucose and labelled ^13^C-glucose (99% C6, Cambridge Isotope Laboratories) were addedat 2.9 and 11.6 mg l^−1^, respectively.After four days the mix of degraded algae and bacteria was extracted from the medium and washed in the same way as described for the axenic algae cultures. Average δ^13^C of the labelled bacteria/algae mixture was 16,883±1,949‰ (equalling 11% ^13^C).

Benthic bacteria were inoculated from coastal sediments and cultivated in a modified M63 medium [38] based on sterilized artificial seawater (salinity of 35 psu). Five g l^−1^ of glucose was added of which half was ^13^C-labelled. After fourdays the bacteria were collected by centrifugation and washed 3-times before storing to remove excess ^13^C-glucose from the bacterial suspension. An average δ^13^C value of 70,991±1,528‰ (equalling 44% ^13^C) was reached.

The four cultured food sources were brought in suspension with artificial seawater, mounted in PVC spheres (4.4 cm diameter, 0.5 cm height, closed at the bottom with parafilm), and kept frozen at −20°C until experimental use. This procedure produced thin frozen slices that could be easily introduced in the experimental tubes while even distribution of the food source was ensured. The carbon content in each slice was determined based on the amount of carbon per litre of culture andequalled 100 mg organic C m^−2^. Due to the lack of information on carbon deposition at the HAUSGARTEN site at the time of the experimental set-up, the carbon concentration was chosen to correspondto the maximum carbon deposition per day calculated for the nearby, seasonally ice-covered East Greenland continental margin (10.9 mg C m^2^ d^−1^, [39]), adapted to the duration of 10 days of the experiment. Meanwhile it has been shown that both areas have comparable seasonal flux patterns and amounts of settled organic matter over the year [40].

### Experimental tube design and sediment preparation

The experimental tubedesign was inspired by Zhou [20]. The tubes weremade from PVC cylinders (4.4 cm diameter) with 500 µm mesh covering the approximately 80% open contour over a length of 15 cm (Figure 1B). The 500 µm mesh was used to provide direct lateral access to nematodes dwelling in the surrounding sediments. The open cylinders were filled with sediment up to 2 cm from the upper mesh rim by pushing them into box corer sediment after the upper 15 cm of sediment was removed. The bottom of the tubes was closed with a conicalcap which easedlater insertion in the sediment.About 3 hours prior to deployment the frozen slices with ^13^C labelled food sources were added on top of the sediment and covered with a few millimetresof sediment before thetubes were taped with parafilm to prevent the sediment from desiccating. The upper lids that closed the tubes had a handle that could easily be manipulated by the ROV *Quest* during deployment and recovery.

### In-situ colonisation/pulse-chase experiment

A total of 15 experimental tubes were prepared: threecontroltubes contained baresediment, and 4-times threetubes contained sediment with one of the initially frozen^13^C-labelled organisms added on top. The tubes were stored in vials filled with seawater and mounted in a rack attached underneath the ROV Quest. At the deep-sea bottom (2475 m water depth) the tubes were handled one by one and inserted in the sediment while taking care that the manipulation would not flush sediment out of the tubes through the mesh. This was visually confirmed during placement and retrieval and later on-board while slicing the sediment. The tubes were randomly distributed in an area covering approximately 1 m^2^ and positioned regularly spaced from one another (±15 cm) (Figure 1C). July 11^th^, the tubes were pushed into the bottom down to the level of the sediment in the tubes, leaving more or less 2 cm of the mesh open for water circulation. After 10 days of incubation, on July 20^th^, the tubes were retrieved undisturbed from the sediment by the ROV *Quest* and brought back to the surface. On board, the upper lid and lower pointedscrew cap were removed and the sediment from the tubes was sliced in 1 cm slices down to 5 cm and one slice from 5 to 10 cm. Sediment slices were stored at −20°C until further analysis in the laboratory.

### Sample processing and analytical procedures

Grain-size distribution from one push core subsample (0–5 cm) and 3 box corer subsamples (15–20 cm) was measured using a Coulter Counter LS 100™ Particle Size Analyser and classified according to Wentworth [41]. Sediments from a second series of subsamples were freeze-dried, homogenized and acidified with diluted HCl (1%) until all carbonates were eliminated, before total organic carbon (TOC) was measured using a Carlo Erba elemental analyser.

Nematodes were extracted from the samples by triple density centrifugation with the colloidal silica polymer LUDOX TM 40 [17] and rinsed with tap water. All nematodes that passed through a 1000 µm mesh and were retained on a 32 µm mesh were counted and handpicked with a fine needle. Due to the low number of colonisers in the experimental tubes, both the community and the ^13^C stable isotope analysis could not be performed on the three replicates from each treatment and control. It was decided to use one sample for nematode community analysis and two samples for measuring δ^13^C values. Nematodes from different centimetre layers had to be combined for the ^13^C analysis to ensure that sufficient biomass was available for EA-IRMS analysis.

All nematodes extracted from the three reference MUC samples, from the four experimental treatments and the control (one replicate each), and from the background box corer samples, were stained with Rose Bengal and transferred to De Grisse I, II and III [42] before being mounted on glass slides. Nematodes were identified to genus level. Adults were distinguished from juveniles based on the development of a vulva and uterus in females and a gonad and spicules in males.Nematode length (L, filiform tail excluded) and maximal width (W) were measured using a Leica DMR compound microscope and Leica LAS 3.3 imaging software. Nematode biomass was then calculated with Andrassy's formula [43]: wet weight (µg) = L (µm)×W^2^ (µm)/1.6 10^6^, and a dry-to-wet-weight ratio of 0.25 was assumed [17].

The nematodes from the two remaining tubes per experimental treatmentand control and from the push core (3-times 200 specimens) were rinsed in MilliQ water and transferred to a drop of MilliQ water in 2.5×6 mm,preglown aluminium cupsat 550°C in order to remove all exogenous organic carbon. Cups with nematodes and ^13^C-labelled organic substrates were oven-dried at 60°C, pinched closed and stored in multi-well Microtitre plates under dry atmospheric conditions until analysis. An elemental analyser-isotope ratio mass spectrometer (EA-IRMS; UC Davis Stable Isotope Facility, California; VUB, Brussels) was used to measure the carbon stable isotope ratios and carbon content. During this procedure a minimal He dilution was applied because ofthe low biomass of the nematode samples. Stable isotope ratios are expressed in the δ notation with Vienna Pee Dee Belemnite (VPDB) as reference standard, and expressed in units of ‰, according to the standard formula*δ* ^13^C = [R_sample_/R_VPDB_−1]×10^3^, where R is the ratio of ^13^C/^12^C (R_sample_ = [(*δ* ^13^C_sample_/1000)+1]×R_VPDB_) and R_VPDB_ is 0.0111802. Label uptake by the nematodes is reflected as enrichment in ^13^C and is presented as Δ*δ* ^13^C (‰), which indicates the increase in δ^13^C of the sample, as compared to its natural background value, and is calculated as Δ*δ* ^13^C (‰) = *δ* ^13^C_sample_−*δ* ^13^C_background_. Hence, positive Δ*δ* ^13^C values indicate that the organismshaveacquired some of the introduced label. Absolute uptake of the label (I) is expressed in µg ^13^C m^−2^ and calculated as I = (F_sample_−F_background_)×S, where F is the ^13^C fraction F = R/(R+1) and S is the total carbon stock (µg C m^−2^) of the nematodes. Biomass-specific respiration rates (R, d^−1^) were based on the de Bovée and Labat formula [44], corrected for in-situ temperature (−0.8°C) assuming *Q*10 is 2: R = 0.0449×*W* ^−0.1456^×exp^ln(*Q*10)/10(*T*-20)^, where *T* is temperature (°C) and *W* is mean individual dry weight (µg C ind^−1^).The individual carbon content was directly inferred from the carbon content measured by the EA-IRMS divided by the number of nematodes in a sample cup.

### Data analysis

Nematode assemblages from the experimental treatments and the reference samples were analysed using univariate and multivariate techniques. One-way ANOVA was applied to assess differences between the univariate variables calculated for each sample (total nematode abundance, number of genera and Shannon-Wiener (H′, log_e_) index). Total nematode abundances were initially logarithmically transformed to fulfil the assumption of homogeneity of variance. The post hoc Tukey HSD test was conducted where the one-way ANOVA obtained significant differences. The nonparametric Mann-Whitney U test was applied when relative abundance data of juveniles did not fulfil the homogeneity of variances test, and was used to test for differences between the colonising and reference nematode assemblages. Non-metric Analysis of Similarities (ANOSIM) was used to test for significant differences between the nematode assemblage structure of the different experimental treatment samplesand the experimental treatment samples versus the reference samples. The analysis was done on Bray-Curtis distances calculated from standardised and log(x+1) transformed abundance data. To visualise the multivariate structure of the nematode genera assemblages, non-metric multi-dimensional scaling ordination (MDS) was performed based on the same Bray-Curtis similarity matrix as used for ANOSIM. The variability among groups of samples was additionally analysed using the multivariate index of dispersion (MID) [45]. To determine the contribution of individual genera to the average Bray-Curtis dissimilarity between the experimental samples and the reference samples, the similarity percentages analysis (SIMPER) was applied to nematode relative abundances.

Nematodes were pooled into biomass and morphometric classes (length, width and length/width) on untransformed geometric scales. Biomass size spectra were created by plotting nematode cumulative relative abundances versus the biomass classes, while for creating the morphometric classes, nematode relative abundance were plotted against geometric classes of length (µm), width (µm) and length/width. All nematode measurements were therefore pooled together in three groups (reference 0–5 cm, colonisation 0–5 cm and colonisation 5–10 cm). A chi-square (*χ^2^*) goodness-of-fit test was performed to test whether the distributions of nematodes in biomass and morphometric classes in the experimental samples differed significantly from the reference samples.

All univariate analyses were performed using the software package STATISTICA 7, considering a confidence level of 0.05 in all tests [46]. The multivariate analyses were carried out using the PRIMER v6 software package [47], [48]. Results are expressed as mean values ± standard deviation of replicates.

## Results

### Sediment characteristics

Sediments from the push core (0–5 cm) and the box corer (15–20 cm) were very fine and characterised by a predominance of silt and clay. The upper 5 cm of the sediment in the push core differed from the 15–20 cm in the box corer by an increase in the clay and silt content(clay: 22% vs. 37±1.2%, silt: 47% vs. 53±2.3%, respectively) and a decrease in the sand fraction (32% vs. 10±1.9%, respectively)with depth. The total organic carbon content was 0.7% and 0.4%, respectively.

### Natural nematode community density and structure

The average density over 5 cm depth in the reference samples was 1223±365 ind.10 cm^−2^. The majority of the nematodes occurred in the top first centimetre with 45±6.7% and gradually decreased downwards. A total of 56 genera were identified in the reference samples. The five dominant genera (relative abundance >5%) altogether accounted for 49% of the total community (*Monhystrella*: 15.7%, *Thalassomonhystera*: 14.6%, *Tricoma*: 6.7%, *Acantholaimus*: 6.2%, *Halalaimus*: 5.9%).

Nematodes were only found occasionally in one of the threesyringe samples taken from the box corer sediment below 15 cm depth (two *Thalassomonhystera*individuals). Their presence can therefore be considered as negligible and not interfering with the colonisation results. From now on the sediment that was used to fill the colonisation tubes will be referred to as ‘azoic sediments’.

### Colonising nematode community density, structure and diversity

**Figure 2:**
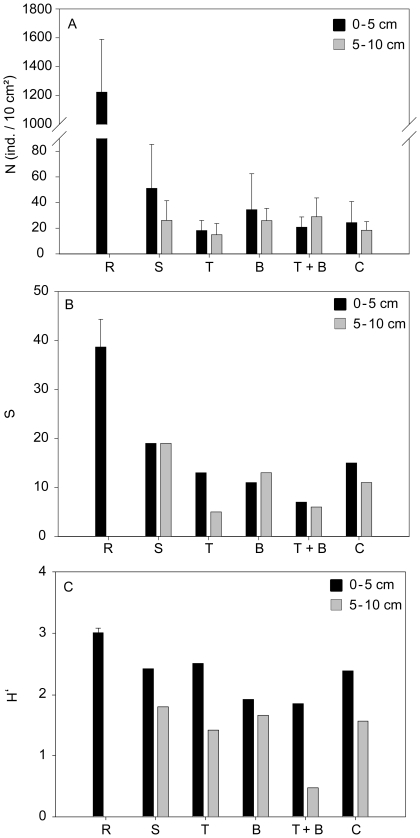
Density (N) and diversity (S, H′) of the nematode assemblages in the reference sediments and all five experimental treatments. Mean and standard deviation where possible for (A) nematode density (N, ind. 10 cm^−2^), (B) number of genera (S) and (C) Shannon-Wiener diversity index (H′, log_e_)in the reference samples (R) and the different experimental treatments(S: *Skeletonema costatum*, T: *Thalassiosira pseudonana*, B: benthic bacteria, T+B: *Thalassiosira pseudonana*+bacteria) and control (C) samples; in the 0–5 cm (black bars) and 5–10 cm (grey bars) sediment layers.

After 10 days of incubation, mean nematode abundance in the first 5 cm of the colonisation cores reached a maximum of 2.13% (51±34 ind. 10 cm^−2^) of the mean nematode abundance from the reference samples (Figure 2A). Because of the generally low and highly variable numbers of nematodes with depth, data from the first 5 cm were grouped for further analysis. As such, nematode abundances did not differ significantly, neither between the different experimental treatments (enriched) and controls (unenriched), nor between the upper and the lower 5 cm within the colonisation tubes (Figure 2A). Compared to the reference, both layers of the colonised sediments contained a higher mean proportion of juveniles, with 74±6% in the 0–5 cm and 75±13% in the 5–10 cm, compared to 58±4% in the reference samples.Only the difference between the top 5 centimetres was significant (p = 0.025).

**Figure 3:**
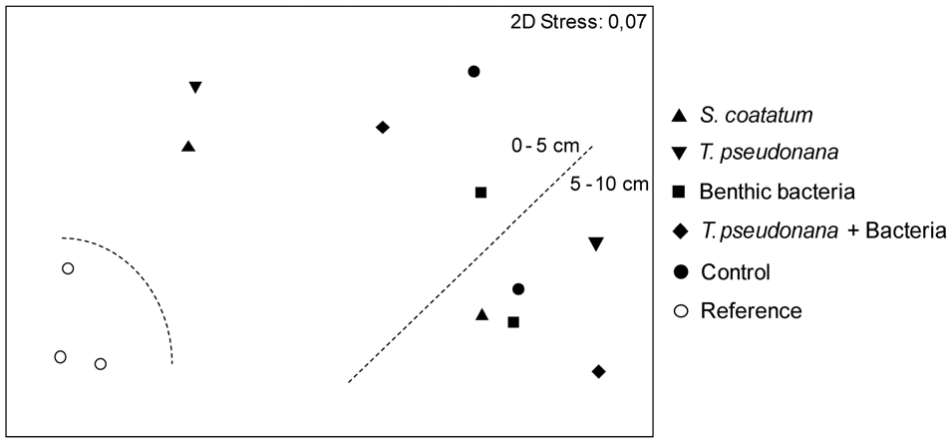
Non-parametric multi-dimensional scaling for nematode assemblages from the references and the experimental controls and enriched treatments (*Sceletonema coatatum*, *Thalassiosira pseudonana*, benthic bacteria and bacteria grown on degrading *T. pseudonana*). The dotted lines separate the samples according to significant ANOSIM grouping results (R≥0.588, p≤0.008).

A pairwise ANOSIM test showed that nematode assemblages in all enriched and unenriched experimental tubes differed in genus composition from the references (0.75≤R≤1), but did not differ from one another (R≤0.25) (Figure 3). Furthermore, a small though significant differentiation in nematode assemblages was found among the experimental tubes according to depth in the sediment (R = 0.588, p = 0.008). The upper and lower 5 cm intervals will further be treated separately to allow comparison with the reference samples which comprise only the upper 5 cm of the sediment. As the different enriched experimental treatments and the unenriched controls did not have a significantly different effect on the nematode assemblage that colonised the tubes, they were considered as one group for further descriptionand analysis of the coloniser assemblage structure. The multivariate index of dispersion (MID) showed that the variability is relatively high among the experimental tubes (0–5 cm: 1.467, 5–10 cm: 0.692) compared with the references (0–5 cm: 0.472).

A total of 41 genera colonised the azoic sediments, of which only 29 were also found in the references. The number of genera found in both the upper and lower 5 cm sediment in the colonisation tubes are also significantly lower than in the references (p<0.0001) (Figure 2B). In the upper 5 cm of the sediment 33 genera were found, while in the lower 5 cm horizon 26 genera were found (7 and 8 genera were not found in the reference samples, respectively). Colonisers that were exclusively found in the colonisation tubes were rare and never occurred with a mean relative abundance higher than 1.5%. Genera that dominate colonisation in the top 5 cm (mean relative abundance >5%) altogether accounted for 67.6% of the total colonisation assemblage (*Sabatieria*: 16.5%, *Thalassomonhystera*: 15.4%, *Leptolaimus*: 12.6%, *Anoplostoma*: 11%, *Dichromadora*: 6%). These four genera were also found in the reference samples (together representing 25%), with only *Thalassomonhystera* as mutual dominant genus. The dominant genera from the reference samples are represented by 21% in the colonising community and genera that can be considered as rare in the reference samples (<1.5% relative abundance) make up 35% of the colonising assemblage in the top 5 cm. In the lower 5 cm of the colonisation tubes, *Sabatieria* was the only dominant genus and accounted for 68.2% of the colonising specimens. Diversity in terms of the Shannon-Wiener index (H′) wasalso significantly higher in the reference samples (3.0±0.08) compared to both depth layers of the colonisationtubes (p≤0.05). Among the colonisation tubes the upper 5 cm of the sediment (2.2±0.31) are characterised by a higher diversity compared to the lower 5 cm (1.4±0.53) (p = 0.017) (Figure 2C).

Results from the SIMPER analysis based on the relative abundance data are complementary to the ANOSIM and MID results and revealed that differences between the references and both depth layers of the colonised azoic sediments were mainly due to higher proportions of *Sabatieria* in the colonised sediments. *Sabatieria* accounted for 12% and 34.6% of the total dissimilarity between the references and the upper 5 cm and the lower 5 cm of the colonisation tubes, respectively. The genus *Anoplostoma* had a relatively high abundance in the upper 5 cm of the colonisation tubes and contributed as such with 10.2% to the dissimilarity with the references. *Monhystrella* and *Thalassomonhystera* both accounted for >5% of the dissimilarity between the reference and the colonised sediments, by occurring in higher relative abundances in the references (Table 1).

**Table 1:** Results from a similarity percentage (SIMPER) analysis, indicating % of similarity and dissimilarity between the nematode assemblages from the reference and the colonisation tubes, with discriminating genera contributing to >5% of the (dis)similarity.

	Reference (0–5 cm)		Colonisation tubes (0–5 cm)		Colonisation tubes (5–10 cm)	
**Reference (0–5 cm)**	**similarity**	**66.5**				
	*Monhystrella*	19.3				
	*Thalassomonhystera*	18.4				
	*Acantholaimus*	7.8				
	*Tricoma*	7.7				
	*Halalaimus*	5.3				
**Colonisationtubes (0–5 cm)**	**dissimilarity:**	**70**	**similarity:**	**43.1**		
	*Sabatieria*	12	*Sabatieria*	27.2		
	*Anoplostoma*	10.2	*Anoplostoma*	24.3		
	*Monhystrella*	9.8	*Dichromadora*	13.7		
	*Thalassomonhystera*	5.9	*Thalassomonhystera*	12.1		
			*Campylaimus*	7.3		
			*Leptolaimus*	6.7		
**Colonisationtubes (5–10 cm)**	**dissimilarity:**	**84.9**	**dissimilarity:**	**64.3**	**similarity:**	**62.2**
	*Sabatieria*	34.6	*Sabatieria*	32.8	*Sabatieria*	86.4
	*Monhystrella*	8.3	*Anoplostoma*	9.4		
	*Thalassomonhystera*	6.8	*Thalassomonhystera*	8.1		

### δ ^13^C stable isotope values

The natural *δ* ^13^C signal derived from 3-times 200 nematode individuals (approximately 10 µg C) from the top 5 cm in the reference push core averaged −18.08±0.3‰. Label uptake by colonisers was traced in bulk nematodes from the upper five and lower five centimetres separately. Due to the low number of nematodes that colonised the experimental tubes, the reliable detection limit of 8–10 µg C was often not reached, despite the higher biomass of a large fraction of the colonisers. Therefore, isotope ratios based on a carbon amount lower than 5 µg C were discarded. The specific ^13^C label uptake (Δ *δ* ^13^C)by the colonising nematodes reached a maximum of 7.31‰ in the treatment where bacteria grown on *Thalassiosira pseudonana* diatoms were offered (Table 2). This equals an absolute uptake (I) of 0.019 µg C tube^−1^ or 0.012% of the added carbon.Based on the formula of de Bovée and Labat [44] for biomass-specific respiration estimates, this corresponds to only 2.8% of carbon respired over 10 days. Although poor replication due to the low number of colonisation hampered statistical analysis, it is clear that in none of the experimental treatments nematodes fed significantly on the labelled potential food sources. This supports the findings on nematode abundances that were not higher in the food treatments compared to the unenriched treatment, indicating that food was not the trigger for nematode colonisation.

**Table 2:** δ^13^C (‰) values of the enriched treatments, based on 10 to 88 nematode individuals per analysis.

		*S. coatatum*		*T. pseudonana*		Benthic bacteria		*T. pseudonana*+Bacteria	
		A	B	A	B	A	B	A	B
**0–5 cm**									
	**µg C:**	6.44	6.47	4.90	/	/	13.53	/	/
	**δ^13^C (‰):**	−15.04	−17.69	−15.40	/	/	−16.30	/	/
									
**5–10 cm**									
	**µg C:**	8.73	9.35	7.78	/	12.29	11.05	5.26	8.52
	**δ^13^C (‰):**	−14.60	−18.83	−19.12	/	−17.72	−17.06	−10.77	−16.14

Replicates are indicated by A and B. Values obtained from less than 5 µg C are left out. Nematode reference δ^13^C values: −18.08±0.3‰ (based on 200 nematode individuals, 3 pseudo-replicates).

### Nematode morphometryand biomass

**Figure 4:**
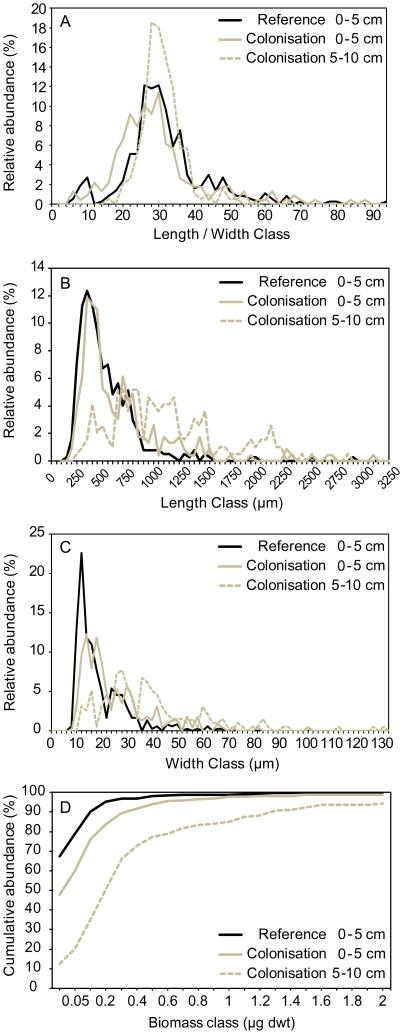
Relative abundance of nematodes in geometric and biomass classes for the reference and experimental colonisation samples, with indication of the respective depth (0–5 cm, 5–10 cm). (A) Length/Width geometric classes (µm), (B) Length geometric classes (µm), (C) Width geometric classes (µm) and (D) Biomass classes (µg dry weight).The relative abundances are based on 372, 228 and 194 individual measurements in the reference, the 0–5 cm colonisation and the 5–10 cm colonisation category, respectively.

Body measurements (length and width) were done on the complete nematode assemblage of the reference and colonisation samples (Figure 4). The distribution of nematode morphometric classes in the reference sediments showed a bimodal shape in case of the length/width ratio, indicating two distinct morphological groups (Figure 4A). These two morphotypes are visualized as two peaks, at L/W ratios of 8–10 and of 24–30, with a distinct minimum at a ratio of 10 to 14, reflecting short, stout nematodes and more slender, on average longer nematodes, respectively. The morphometric class distribution in both 5 cm depth layers of the colonised sediments shifted from the reference distribution in a different way but lacked this distinct group of short, stout morphotypes, represented by the genera *Desmoscolex* and *Tricoma*, in common. In the upper 5 cm of the colonised sediments there was a relatively large shift towards greater body width compared to the shift towards greater body length, resulting in more plump nematodes (Figure 4A–C). The more equally spread distribution in length and width of the colonisersin the 5–10 cm sediment depth led to a narrower spectrum and indicates more slender morphotypes are present (Figure 4A–C).In the reference samples, 20% of the nematodes have a L/W ratio <24, while in the experimental samples the shift is clear towards 37% in the upper 5 cm and towards 10% in the lower 5 cm of the sediment. Nematodes assemblages from the 5–10 cm colonised sediments were characterized by a greater body length and body width compared to both the reference and upper 5 cm of colonised sediments (*χ* ^2^ test, p<0.0001).

In the reference samples, 95% of the nematode assemblage consisted of individuals with a biomass of less than 0.2 µg dry weight (Figure 4D). This biomass class represented 84% in the upper 5 cm and only 51% in the 5–10 cm in the colonised sediment. A chi-squared (*χ* ^2^) analysis showed that colonisation of the 5–10 cm sediments was characterized by nematodes with higher biomass, compared to the upper 5 cm of the colonised sediments and the reference sediments (p<0.0001).

## Discussion

Studies on the response of deep-sea nematode communities to organic matter pulses generallyfocussed on changes in species composition and biomass patterns, or feeding ecology by means of simulated phytodetritus fluxes with ^13^C-labelled algae or bacteria. Our study differs by investigating the reaction of deep-sea nematodes on ^13^C-labelled organic matter in terms of colonisation and uptake. Although conducting experiments in situ in the poorly accessible deep sea, using ROV technology, renders very precise control over the experiment, it also comes at a cost. The limited storage space on the ROV, in combination with limited ship time, restricted replication of the experimental treatmentsand only allowed sampling at one moment in time. Replication was further restricted by the low number of colonising nematodes. Nonetheless, our experimental set up allowed simulating disturbance and the arrival of different types of organic matter in situ, with minimal manipulation or influence on the natural nematode community.

### Colonisation rates

The infaunal colonisation of azoic sediments observed in our experiment confirms the first hypothesis (H_1_) that deep-sea nematodes can actively colonise open patches. It affirms the findings of Gallucci et al. [28] that deep-sea nematodes have the ability to respond within a time frame of a few days (or less). The colonisation rates in the upper 5 cm of our experimental tubes(max. 51 ind. 10 cm^−2^, i.e. 2% of the reference) arehowever lower than reportedfor infaunal migration of coastal nematodes, where nematodes relative densities reached 4–36% after 2 days and 19–31% after 29 days [31]; 4–7% after 10 days [20]; and 32–90%after 14 days [29] of the reference nematode abundances. Gallucci et al. [28] used a comparable setup with the upper 5 cm sediment from 1300 m of water depth in an on-board microcosm experiment and found colonisation rates of approximately 5–20% after 9 days, without significant difference after 17 days. These rates are similar to or at the lower end of the rates found for coastal sediments, but higher than reported in the present study. In case where food was added to the sediment, the higher concentrations of added organic carbon in the experiment of Gallucci et al. [28] might have induced higher colonisation rates (see below) [20]. Nonetheless,the colonisation rates in the unenriched controls in this study are also comparatively low. It should be considered that the use of deeper sediment layers to fill the colonisation tubes might have introduced a barrier or perhaps less favourable conditions for nematodes to colonise. Different sediment types had an effect on the colonisation rates of nematodes from an intertidal coastal station [29]. In our study the colonised sediments contained a higher fraction of clay and silt, but perhaps also other sediment parameters that we did not measure may have played a role (e.g. porosity, pH, O_2_ concentration). Another factor with unknown impact is the effect of decompression prior to the laboratory experiment of Gallucci et al. [28]. These uncertainties withhold us from supporting the debated suggestion on slower colonisation rates in the deep sea [5], [49].

When compared to the upper 5 cm of the colonised sediments, nematodes occurred in equal abundances and number of genera, below 5 cm in all experimental treatments.The nematode assemblage that colonised the top 5 cm of the experimental tubes was however more diverse due to the dominance (with 68%) of *Sabatieria* in the lower 5 cm of the colonisation tubes. Overall, the total number of genera that colonised the azoic sediments in our short-term experiment was relatively high (41 genera) and only slightly lower compared to the reference situation (56 genera). Together with the high variability between the colonising assemblages, our results confirm that early deep-sea colonisation of disturbed patches is a highly localised process, with reproducible abundance and diversity patterns, but a relatively poor predictability of the community composition [28].

### The role of organic matter in the colonisation process

Nematode assemblages colonised the azoic sediments in the enriched and the unenriched experimental tubes in equal numbers and with similar diversity, irrespective of the presence or the type of organic matter added. Based on these observations the second hypothesis (H_2_), that postulated that colonisation rates are enhanced by the presence of food and led to higher abundances and number of genera, isrejected. This is in contrast with the experimental results of Gallucci et al. [28] who observed higher nematode abundances and number of nematode speciesin diatom enriched sediments compared to unenriched sediments after 9 days in a microcosm experiment performed at a shallower station at the Arctic HAUSGARTEN site. The four times higher nematode abundance in enriched compared to the unenriched sediments observed by Gallucci et al. [28] might be the result of applying higher concentration of organic matter compared to this study (1 g vs. 100 mg organic C m^−2^, respectively).The positive effect of higher concentrations of organic matter was found before in a colonisation experiment in a mangrove [20]. As a result of leaf litter addition significantly higher relative nematode numberscompared to the controls appeared after 30 days and showed a positive response to different levels of leaf litter addition after 60 days.This response was however mainly due to a single opportunistic,bacterivorous species (*Diplolaimella*sp.)that represented up to 84% of the colonising assemblage, and was otherwise rare in unenriched and ambient mangrove sediments.In the study by Gallucci et al. [28],not one but several genera that were rare or undetected in the natural sediments all together dominated colonisation of defaunated sediments, indicating a different reaction. In contrast to [28], the rare and undetected genera from the reference samples in our study only represented 35% of the relative abundance of the colonising assemblage.If the suggested slower colonisation rates in the deepsea hold true, our findings might reflect an earlier stage in the colonisation process compared to [28].

Additionally, we could verify that the colonising nematodes did not feed significantly on the different types of labelled food added to the experimentaltubes. This also means a rejection of the third and fourth hypothesis (H_3_ and H_4_). While food is generally thought of as an attracting factor for free-living marine nematodes (e.g. [45]–[52]) in some cases it seems as if certain food varieties or stages of decomposition have a repellent or indifferent effect on some communities and species [20], [50], [53]. The indifferent effect ofthe presence of pelagic diatoms as observed in our experiment was also found under experimental conditions by Ullberg and Ólafsson [54]. They suggested that nematodes from the shallow coastal station they sampled and incubated preferred in-situ growing diatoms above pelagic diatoms as they more closely resemble the natural circumstances. However, by using thecosmopolitan pelagic diatoms, benthic bacteria and bacteria grown on degrading pelagic diatoms (detritus) we covered the most relevant range of potential food sources that deep-sea nematodes have access to. Bacterial biomass is one to two orders of magnitude higher than nematode biomass in ocean basins worldwide [35] and, according to respiration estimations of a typical deep-sea nematode community, a theoretically non-limited potential food source [55]. Nonetheless, the results from this colonisation experiment and from a ^13^C-tracer experiment that directly labelled the natural deep-sea bacterial community at 1280 m depth the HAUSGARTEN site, both suggest that on a short term bacterial carbon is of no substantial importance in the diet of the deep-sea nematode community [55]. On the other hand, organic matter enrichment by means of diatoms seemed especially relevant based on the fact thatthey are among the organisms that dominate the POM flux derived from phytoplankton blooms following the retreat of winter seaice at high latitudes (maxima in May–June and August–September) [40]. Evidence of high-quality detritus rapidly reaching the deep-sea floor has been reported for numerous deep-sea sites (e.g. [56]–[58]). Nevertheless, in terms of uptake, the results of this study support other recent ^13^C stable isotope tracer experiments in deep-sea sediments, all indicating a limited contribution of nematodes to the short-term processing of labelledphytodetritus [12], [13], [15], [16], [59], [60].

One plausible explanation is that the experiment was performed at the same time or after a natural phytodetritus flux settled to the sea floor and nematodes were surrounded by or already processed a great amount of detritus, leaving them indifferent to the food we applied.Additionally or alternatively they might have a very specific preference for a certain component of the sediment or detritus flux that we did not address (e.g. coccolithophorids, pennate diatoms, ciliates, protists like tintinnids, foraminifera, radiolaria or acantharia [40]). Forest et al. [61] and Bauerfeind et al. [40] however, recently illustrated that not more than 2% of the photosynthetically-produced carbon in the Fram Strait may reach ∼300 m water depth due to a high degree of recycling within the upper water column. Fresh diatoms were negligible in the sediment trap samples and most of them were degraded frustules with no plasma content [61]. These results indicate that coupling between pelagic-benthic fluxes might be overestimated in high-north latitudes. Nonetheless, high primary production at the ice edge results in high phytodetritusconcentrations at the sea floor [62] and nematode densities are correlated with water depthand pigment concentrations in the sediment at the HAUSGARTEN site [63]. Therefore, an alternative, though speculative,explanation for the lack of uptake might be that the combination ofseasonal detritus fluxes, low-temperature induced slow nematode growth, long life span and low maintenance costs [19], and low-temperature reduced microbial respiration at low concentrations of organic matter [64], lead to an accumulation of labile organic material in Arctic deep-sea sediments.Similar to what is suggested for Antarctic shelf sediments by Mincks et al. [65] and Smith et al. [66] this could result in a persistent ‘food bank’ for benthic detrivores over long time scales relative to the seasonality of POC deposition. Additionally, if the success of deep-sea nematodes lies partly on trophic specialisation that implies passive deposit feeding rather than active selection of food particles, as suggested by Giere [19], nematodes might be less limited in food than is expected till now. This could enforce the possibility that the deep-sea nematode colonisation processes in the Arctic area are not mainly driven by food-related features, as observed in our experiment, but that it is rather the result of attraction by biogeochemical cues that were unaccounted for or ‘random walk’ behaviour (whether or not driven by the search for a mating partner). However, the lack of data that cover seasonal variability in concentrations of labile organic matter (reflected in e.g. chlorophyll a, enzymatically hydrolysable amino acids, etc.), as well as concentrations of POC and stable isotope ratios (δ^13^C and δ^15^N) of both settling POM and surface sediments at the Arctic ice margin, does not enableus to verify if the sediment has a buffering effect against water column variability, and the labile organic matter persists year-round, or not.

### Colonising efficiency

The nematode assemblages that colonised the azoic sediments differed significantly from the natural nematode assemblage. Some genera colonised more efficiently, thus supporting the fifth hypothesis (H_5_) that active migration is species-specific. The colonisation success of certain *Sabatieria* and*Leptolaimus*species for example was emphasised by Gallucci et al. [28], Ullberg and Ólafsson [67] and Schratzberger et al. [29], and is confirmed here. These observations indicate that in shallow- and deep-water environments species association upon initial colonisation is not simply the result of random processes, but determined by species characteristics such as their motility and colonisation ability [68].While in the upper 5 cm of the colonised sedimentsfive dominant genera (*Sabatieria*, *Thalassomonhystera*, *Leptolaimus*, *Anoplostoma* and *Dichromadora*) accounted for 68% of all nematodes encountered, *Sabatieria* alone dominated with 68% in the lower 5 cm. The success of some *Sabatieria* specieshas been attributed to theirhigh tolerance to anoxia, relatively big size (adult average length in this study: 1918±285 µm) and higher mobility which enables them to move through compacted subsurface sediments and easily access oxygen in the upper layers [69]. While members of the genus are found in muddy sediments that are anoxic from just a few millimetres below the sediment surface, *Sabatieria* also occurs deeper down in more oxygenated sediments, in the vicinity of the redox-potential-discontinuity layer [70]–[72]. Sachs et al. [73] reported oxygen penetration at the central HAUSGARTEN station (2500 m water depth)down to 15 cm.Therefore it is feasible that *Sabatieria*livesbelow 5 cm where oxygen and also organic matter are limited, and regularly migrates upwards to access food. This was demonstrated for *Sabatieria* at a subtidal station in the southern North Sea by means of natural stable isotope signatures [74].Unfortunately, reference data of the nematode community for the HAUSGARTEN site are restricted to the upper 5 cm of the sediment.Nonetheless, *Sabatieria* is demonstrated to be among the most efficient colonisers, especially of deeper sediment layers. *Thalassomonhystera* on the other hand, belongs to the family Monhysteridae, whose reproductive rates are generally higher and development rates faster than formost other marine nematodes [17], [75].Having the characteristics of an r-strategist, it is curious that *Thalassomonhystera*'s dominance prevailed in later successional stages. In contrast, other dominant genera in the reference samples: *Monhystrella* (family Monhysteridae), *Acantholaimus* and*Halalaimus*, were rarely encountered in the experimental tubes. These genera are characterised by a long filiform tail, which was suggested to be typical for a hemi-sessile life strategy [76]. Although the size of *Acantholaimus* and *Halalaimus* not necessarily implieslimited active migration ability, as is suggested for the very small *Monhystrella* genus, their response to disturbance may as well involve a different strategy (e.g. high reproductive rates) and rely less on motility [28].The remaining 32% of the total nematode assemblage that colonised the azoic sediments counted 36 genera of which twelve genera were exclusively found in the colonisation tubes, never with a relative abundance higher than 1.5%. The absence of these rare genera from the reference samples might partly be a result of undersampling, as three reference (pseudo-)replicates underestimate the natural variability and genus richness of nematode assemblages on the small local scale of the experiment. This is confirmed by the fact that 7 of these 12 genera were encountered by Hoste [77] in a 5 year time-series study across a depth gradient at the HAUSGARTEN site.On the other hand, the fact that 35% of the colonising assemblage are rare or absent in the reference samples might also support and argue that a quick response to open space and/or organic matter deposition is an important life strategy to counterbalance local extinctions and inhabit a patchily disturbed environment [28].

The distribution of nematode morphometric classes (L/W) in the reference sediments showed a bimodal shape. This is a typical and persistent feature found in nematode communities in continental slope areas around the world, indicating two distinct morphological groups [69].In the colonised sediments, the size spectrum shifted towards thicker nematodesin the upper 5 cm and longer and wider nematodes in the lower 5 cm, confirming the fifth hypothesis (H_5_) and the experimental findings of Gallucci et al. [28] that active migration is dependent on nematode size. Our findings suggest two potential strategies. The thicker nematodes found in the upper 5 cm of the colonised sediments might be genera that allocate a larger amount of food to storage products rather than to structural growth. This could foresee them with the advantage of remaining an increased amount of time without food, depending on their stored reserve products [69]. On the contrary, the bigger individuals found in the lower 5 cm of the sediment most probably and perhaps additionally have the advantage of a high mobility, which provides accessibility to food and open space,and enables exchange between hypoxic subsurface and oxic surface layers on a short notice. The shift in nematode size towards bigger colonisersalso co-occurs with a shift in developmental stages towards more juveniles in the colonising assemblage. This confirms the suggestion of Soetaert et al. [69] that if bigger nematodes have a higher age at maturity, the nematode communities that are, on average, composed of larger species would be more dominated by juveniles. This statement is based on the assumption that smaller-sized species that have higher growth rates (biomass increase [78]) also have reduced age at first breedingin the deep sea [69].

### Prospects

Caution should be taken when extrapolating the results to situations of medium- to large-scale disturbances.Not only can colonisation processes vary markedly between patches of different sizes [79] or disturbance intensity, the process will also be more complex when it is influenced by water column processes such as bottom currents or sediment upwelling transporting nematodes [54], or macrofaunal interstitial migration and larval settlement. This demonstrates that the understanding of local dynamics in the deep sea is still in its infancy. Meanwhile, the idea and associated modelling efforts arose on local communities being embedded in a metacommunity, where it is likely that various spatial dynamics alter local community responses that feed back to alter features of regional biota [80]. Therefore, if we want to (1) make progress in understanding deep-sea community ecology, (2) implement local dynamics in metacommunity models and (3) anticipate the impact of acute disturbances in the deep sea, it is required to gather autoecological information and perform prolonged in-situ experimental incubations with repeated sampling over time to follow up further successional stages and complete recovery time.

### Conclusion

This studydemonstrated that deep-sea nematodes actively colonised azoic sediments within a time frame of 10 days, regardless the presence or the type of organic matter that was added.The fact that organic matter did not function as an attractant was confirmed by the absence of notable ^13^C assimilation by the colonising nematodes. Colonisation by deep-sea nematodes appears to be a process yielding reproducible abundance and diversity patterns, with certain taxa showing more efficiency, partly based on body shape characteristicssuch as size and motility. Colonisation by a relatively large number of genera that were rare or undetected in reference sediments also suggests that small-scale disturbance contributes to their persistence in deep-sea sediments. Togetherwith the high variability between the colonising nematode assemblages, theseresults lend experimental support to the existence of a spatio-temporal mosaic that emerges from highly localised, both stochastic and deterministic community dynamics.

## References

[1] Levin LA, Etter RJ, Rex MA, Gooday AJ, Smith CR (2001). Environmental influences on regional deep-sea species
diversity.. Annu Rev Ecol Syst.

[2] Thistle D (1981). Natural physical disturbances and communities of marine soft
bottoms.. Mar Ecol Prog Ser.

[3] Johnson RG (1970). Variations in diversity within benthic marine
communities.. Am Nat.

[4] Grassle JF, Sanders HL (1973). Life histories and the role of disturbance.. Deep-Sea Res.

[5] Gage JD (1991). Biological rates in the deep sea: a perspective from studies on
processes in the benthic boundary layer.. Rev Aquat Sci.

[6] Gooday AJ (2002). Biological responses to seasonally varying fluxes of organic
matter to the seafloor: a review.. J Oceanogr.

[7] Ruhl HA, Ellena JA, Smith KL (2008). Connections between climate, food limitation, and carbon cycling
in abyssal sediment communities.. Proc Nat Acad Sci USA.

[8] Smith CR (1986). Nekton falls, low intensity disturbance and community structure
of infaunal benthos in the deep sea.. J Mar Res.

[9] Grassle JF, Morse-Porteous LS (1987). Macrofaunal colonization of disturbed deep-sea environments and
the structure of deep-sea benthic communities.. Deep-Sea Res.

[10] Soltwedel T (1997). Temporalvariabilities in benthic activity and biomass on the
western European continental margin.. Oceanol Acta.

[11] Levin LA, Blair NE, Martin CM, DeMaster DJ, Plaia G (1999). Macrofaunal processing of phytodetritus at two sites on the
Carolina margin: in situ experiments using ^13^C-labeled
diatoms.. Mar Ecol Prog Ser.

[12] Moodley L, Middelburg JJ, Boschker HTS, Duineveld GCA, Pel R (2002). Bacteria and Foraminifera: key players in a short-term deep-sea
benthic response to phytodetritus.. Mar Ecol Prog Ser.

[13] Ingels J, Billett DSM, Van Gaever S, Vanreusel A (2010). An insight into the feeding ecology of deep-sea canyon nematodes
- Results from field observations and the first
*in-situ*
^13^C feeding experiment in the
Nazaré Canyon.. J Exp Mar Biol Ecol.

[14] Ingels J, Van Den Driessche P, De Mesel I, Vanhove S, Moens T (2010). Preferred use of bacteria over phytoplankton by deep-sea
nematodes in polar regions.. Mar Ecol Prog Ser.

[15] Nomaki H, Heinz T, Nakatsuka T, Shimanaga M, Kitazato H (2005). Species-specific ingestion of organic carbon by deep-sea benthic
foraminifera and meiobenthos: In situ tracer experiments.. Limnol Oceanogr.

[16] Sweetman AK, Witte U (2008). Response of an abyssal macrofaunal community to a phytodetrital
pulse.. Mar Ecol Prog Ser.

[17] Heip CHR, Vincx M, Vranken G (1985). The ecology of marine nematodes.. Oceanogr Mar Biol Annu Rev.

[18] Lambshead PJD (1993). Recent development in marine benthic diversity
research.. Oceanis.

[19] Giere O (2009). Meiobenthology: the microscopic motile fauna of aquatic
sediments..

[20] Zhou H (2001). Effects of leaf litter addition on meiofaunal colonization of
azoic sediments in a subtropical mangrove in Hong Kong.. J Exp Mar Biol Ecol.

[21] Menot L, Crassous P, Desbruyères D, Galéron J, Khripounoff A (2009). Colonization patterns along the equatorial West African margin:
implications for functioning and diversity maintenance of bathyal and
abyssal communities.. Deep-Sea Res II.

[22] Snelgrove PVR, Grassle JF, Petrecca RF (1992). The role of food patches in maintaining high deep-sea diversity:
field experiments with hydrodynamically unbiased colonization
trays.. Limnol Oceanogr.

[23] Quijón PA, Kelly MC, Snelgrove PVR (2008). The role of sinking phytodetritus in structuring shallow-water
benthic communities.. J Exp Mar Biol Ecol.

[24] Billheimer LE, Coull BC (1988). Recolonization of meiobenthos into juvenile spot (Pisces) feeding
pits.. Estuar Coast Shelf Sci.

[25] Savidge WB, Taghon GL (1988). Passive and active components of colonization following two types
of disturbance on intertidal sandflat.. J Exp Mar Biol Ecol.

[26] Sherman KM, Coull BC (1980). The response of meiofauna to sediment
disturbance.. J Exp Mar Biol Ecol.

[27] Tita G, Vincx M, Desrosiers G (1999). Size spectra, body width and morphotypes of intertidal nematodes:
an ecological interpretation.. J Mar Biol Assoc UK.

[28] Gallucci F, Moens T, Vanreusel A, Fonseca G (2008). Active colonisation of disturbed sediments by deep-sea nematodes:
evidence for the patch mosaic model.. Mar Ecol Prog Ser.

[29] Schratzberger M, Whomersley P, Warr K, Bolam SG, Rees HL (2004). Colonisation of various types of sediment by estuarine nematodes
via lateral infaunal migration: a laboratory study.. Mar Biol.

[30] Alongi DM, Boesch DF, Diaz RJ (1983). Colonization of meiobenthos in oil-contaminated subtidal sands in
the Lower Chesapeake Bay.. Mar Biol.

[31] Chandler GT, Fleeger JW (1983). Meiofaunal colonization of azoic estuarine sediment in Louisiana:
mechanisms of dispersal.. J Exp Mar Biol Ecol.

[32] Ólafsson E, Moore CG (1990). Control of meiobenthic abundance by macroepifauna in a subtidal
muddy habitat.. Mar Ecol Prog Ser.

[33] Sherman KM, Reidenauer JA, Thistle D, Meeter D (1983). Role of natural disturbance in an assemblage of marine
free-living nematodes.. Mar Ecol Prog Ser.

[34] Widbom B (1983). Colonization of azoic sediment by sublittoralmeiofauna in Gullmar
Fjord - Swedish West coast.. Oceanol Acta Spec.

[35] Rex MA, Etter RJ, Morris JS, Crouse J, McClain CR (2006). Global bathymetric patterns of standing stock and body size in
the deep-sea benthos.. Mar Ecol Prog Ser.

[36] Soltwedel T, Bauerfeind E, Bergmann M, Budaeva N, Hoste E (2005). HAUSGARTEN: multidisciplinary investigations at a deep-sea
longterm observatory in the Arctic Ocean.. Oceanography.

[37] Guillard RRL, Smith WL, Chanley MH (1975). Culture of phytoplankton for feeding marine
invertebrates.. Culture of marine invertebrate animals.

[38] Miller JH (1972). Experiments in molecular genetics.

[39] Sauter EJ, Schlüter M, Suess E (2001). Organic carbon flux and remineralization in surface sediments
from the northern North Atlantic derived from pore-water oxygen
microprofiles.. Deep-Sea Res I.

[40] Bauerfeind E, Nöthig E-M, Beszczynska A, Fahl K, Kaleschke L (2009). Particle sedimentation patterns in the eastern Fram Strait during
2000–2005: Results from the Arctic long-term observatory
HAUSGARTEN.. Deep-Sea Res I.

[41] Wenthworth CK (1922). The Wenthworth scale of grain size for sediments.. J Geol.

[42] Seinhorst JW (1959). A rapid method for the transfer of nematodes from fixative to
anhydrous glycerine.. Nematologica.

[43] Andrassy I (1956). The determination of volume and weight of
nematodes.. Acta Zool Hung.

[44] deBovée F, Labat PH (1993). A simulation model of a deep meiobenthic compartment: a
preliminary approach.. Mar Ecol.

[45] Warwick RM, Clarke KR (1993). Increased variability as a symptom of stress in marine
communities.. J Exp Mar Biol Ecol.

[46] StatSoft Inc (2010). Electronic Statistics Textbook.. http://www.statsoft.com/textbook/.

[47] Clarke KR, Gorley RN (2001). PRIMER v.5. User manual..

[48] Clarke KR, Warwick RM (2001). Changes in marine communities: an approach to statistical
analysis and interpretation..

[49] Grassle JF (1977). Slowrecolonization of deep-sea sediments.. Nature.

[50] Lee J, Tietjen J, Mastropaolo C, Rubin H (1977). Food quality and the heterogeneous spatial distribution of
meiofauna.. Helgol Wiss Meeresunters.

[51] Neira C, Sellanes J, Levin LA, Arntz WE (2001). Meiofaunal distributions on the Peru margin: relationship to
oxygen and organic matter availability.. Deep-Sea Res.

[52] Höckelmann C, Moens T, Juttner F (2004). Odor compounds from cyanobacterial biofilms acting as attractants
and repellents for free-living nematodes.. Limnol Oceanogr.

[53] Gooday AJ, Pfannkuche O, Lambshead PJD (1996). An apparent lack of response by metazoan meiofauna to
phytodetritus deposition in the bathyal North-Eastern
Atlantic.. J Mar Ass UK.

[54] Ullberg J, Ólafsson E (2003). Free-living marine nematodes actively choose habitat when
descending from the water column.. Mar Ecol Prog Ser.

[55] Guilini K, Van Oevelen D, Soetaert K, Middelburg JJ, Vanreusel A (2010). Nutritional importance of benthic bacteria for deep-sea nematodes
from the Arctic ice margin: Results of an isotope tracer
experiment.. Limnol Oceanogr.

[56] Bealieu SE (2002). Accumulation and fate of phytodetritus on the sea
floor.. Oceanogr Mar Biol Annu Rev.

[57] Billett DSM, Lampitt RS, Rice AL, Mantoura RFC (1983). Seasonal sedimentation of phytoplankton to the deep-sea
benthos.. Nature.

[58] Lampitt RS (1985). Evidence for the seasonal deposition of detritus to the deep-sea
floor and its subsequent resuspension.. Deep-Sea Res.

[59] Moens T, Vanhove S, De Mesel I, Keleman B, Janssens T (2007). Carbon sources of Antarctic nematodes as revealed by natural
carbon isotope ratios and a pulse-chase experiment.. Polar Biol.

[60] Witte U, Wenzhöfer F, Sommer S, Boetius A, Heinz P (2003). In situ experimental evidence of the fate of a phytodetritus
pulse at the abyssal sea floor.. Nature.

[61] Forest A, Wassmann P, Slagstad D, Bauerfeind E, Nöthig EM (2010). Relationships between primary production and vertical particle
export at the Atlantic-Arctic boundary (Fram Strait,
HAUSGARTEN).. Polar.

[62] Schewe I, Soltwedel T (2003). Benthic response to ice-edge-induced particle flux in the Arctic
Ocean.. Polar Biol.

[63] Hoste E, Vanhove S, Schewe I, Soltwedel T, Vanreusel A (2007). Spatial and temporal variations in deep-sea meiofauna assemblages
in the Marginal Ice Zone of the Arctic Ocean.. Deep-Sea Res I.

[64] Nedwell DB (1999). Effect of low temperature on microbial growth: lowered affinity
for substrates limits growth at low temperature.. FEMS Microbiol Ecol.

[65] Mincks SL, Smith CR, DeMaster DJ (2005). Persistence of labile organic matter and microbial biomass in
Antarctic shelf sediments: evidence of a sediment ‘food
bank’.. Mar Ecol Prog Ser.

[66] Smith CR, Mincks SL, DeMaster DJ (2006). A synthesis of bentho-pelagic coupling on the Antarctic shelf:
food banks, ecosystem inertia and global climate change.. Deep-Sea Res II.

[67] Ullberg J, Ólafsson E (2003). Effects of biological disturbance by
*Monoporeiaaffinis* (Amphipoda) on small-scale migration
of marine nematodes in low-energy soft sediments.. Mar Biol.

[68] Horn HS, Mary RM (1981). Succession.. Theoretical ecology: principles and applications.

[69] Soetaert K, Muthumbi A, Heip CHR (2002). Size and shape of ocean margin nematodes: morphological diversity
and depth-related patterns.. Mar Ecol Prog Ser.

[70] Vanreusel A (1990). Ecology of the free-living marine nematodes from the Voordelta
(Southern Bight of the North Sea). I. Species composition and structure of
the nematode communities.. Cah Biol Mar.

[71] Vincx M, Meire P, Heip C (1990). The distribution of nematode communities in the Southern bight of
the North Sea.. Cah Biol Mar.

[72] Hendelberg M, Jensen P (1993). Vertical distribution of the nematode fauna in a coastal sediment
influenced by seasonal hypoxia in bottom water.. Ophelia.

[73] Sachs O, Sauter EJ, Schlüter M, Rutgers van der Loeff MM, Jerosch K (2009). Benthic organic carbon flux and oxygen penetration reflect
different plankton provinces in the Southern Ocean.. Deep-Sea Res I.

[74] Franco MA, Soetaert K, Van Oevelen D, Van Gansbeke D, Costa MJ (2008). Density, vertical distribution and trophic responses of metazoan
meiobenthos to phytoplankton deposition in contrasting sediment
types.. Mar Ecol Prog Ser.

[75] Bongers T, Alkemanade R, Yeates GW (1991). Interpretation of disturbance-induced maturity decrease in marine
nematode assemblages by means of Maturity Index.. Mar Ecol Prog Ser.

[76] Riemann F (1974). Onhemisessile nematodes with flagelliform tails living in marine
soft bottoms and on microtubes found in deep sea sediments.. Mikrofauna Meeresboden.

[77] Hoste E (2006). Temporal and spatial variability in dee-sea meiobenthic
communities from the Arctic Marginal Ice Zone..

[78] Peters RH (1983). The ecological implications of body size.

[79] Smith CR, Brumsickle SJ (1989). The effects of patch size and substrata isolation on colonization
modes and rates in an intertidal sediment.. Limnol Oceanogr.

[80] Leibold MA, Holyoak M, Mouquet N, Amarasekare P, Chase JM (2004). The metacommunity concept: a framework for multi-scale community
ecology.. Ecol Lett.

